# Suppressor of cytokine signaling 3 plays an important role in porcine circovirus type 2 subclinical infection by downregulating proinflammatory responses

**DOI:** 10.1038/srep32538

**Published:** 2016-09-01

**Authors:** Xuejiao Zhu, Juan Bai, Panrao Liu, Xianwei Wang, Ping Jiang

**Affiliations:** 1 Key Laboratory of Animal Diseases Diagnostic and Immunology, Ministry of Agriculture, College of Veterinary Medicine, Nanjing Agricultural University, Nanjing 210095, China; 2 Jiangsu Co-innovation Center for Prevention and Control of Important Animal Infectious Diseases and Zoonoses, Yangzhou, China

## Abstract

Porcine circovirus type 2 (PCV2) causes porcine circovirus-associated diseases and usually evokes a subclinical infection, without any obvious symptoms, in pigs. It remains unclear how PCV2 leads to a subclinical infection. In this study, we found that peripheral blood mononuclear cells (PBMCs) from PCV2-challenged piglets with no significant clinical symptoms exhibited increased expression of suppressor of cytokine signaling (SOCS) 3, but no significant changes in the expression of the proinflammatory cytokines interleukin (IL)-6 and tumor necrosis factor (TNF)-α; this differed from piglets that displayed significant clinical symptoms. IL-6- and TNF-α-mediated signalings were inhibited in PBMCs from subclinical piglets. Elevated SOCS3 levels inhibited IL-6- and TNF-α-mediated NF-kappa-B inhibitor alpha degradation in PBMCs and PK-15 cells. SOCS3 production was also increased in PCV2-infected PK-15 porcine kidney cells, and IL-6 and TNF-α production that was induced by PCV2 in PK-15 cells was significantly increased when SOCS3 was silenced by a small interfering RNA. SOCS3 interacted with signal transducer and activator of transcription 3 and TNF-associated receptor-associated factor 2, suggesting mechanisms by which SOCS3 inhibits IL-6 and TNF-α signaling. We conclude that SOCS3 plays an important role in PCV2 subclinical infection by suppressing inflammatory responses in primary immune cells.

Porcine circovirus type 2 (PCV2) is a single-stranded, non-enveloped DNA virus that is a member of the *Circoviridae* family^1^,^2^. The clinical symptoms caused by PCV2 range from subclinical to several virus-associated syndromes, such as post-weaning multisystemic wasting syndrome (PMWS)^1^,^3^,^4^, necrotizing lymphadenitis^5^, acute pulmonary edema^6^, and reproductive disorder and granulomatous enteritis^7^,^8^,^9^, which are collectively named porcine circovirus diseases. However, PCV2 often triggers subclinical infections, with the infected pigs showing no apparent symptoms and signs. PCV2 preferentially targets lymphoid tissues, which leads to lymphoid depletion and immunosuppression in pigs. PCV2 resides in certain immune cells, such as macrophages and dendritic cells, and modulates their functions^10^. Peripheral blood mononuclear cells (PBMCs) from PCV2-infected piglets stimulated with concanavalin A, or from piglets with PMWS, show increased interleukin (IL)-2, IL-4, IL-6, and IL-10 levels^10^,^11^. The upregulation of IL-10 and proinflammatory cytokines in infected pigs may contribute to pathogenesis. However, until recently, knowledge of the pathogenicity of PCV2 has been limited, and it remains unclear how PCV2 leads to a subclinical infection.

The suppressor of cytokine signaling (SOCS) family consists of cytokine-inducible negative regulators of cytokine signaling. Some cytokines, including IL-6, interferon (IFN)-γ, and lipopolysaccharide are capable of inducing the SOCS family and its associated negative feedback of cytokines^12^,^13^,^14^. SOCS3 inhibits the signaling of IL-6, granulocyte colony-stimulating factor, and leptin by binding to their respective receptors. SOCS3 interacts with glycoprotein (GP) 130^15^, Janus kinase (JAK) 2^16^,^17^,^18^, erythropoietin receptor^19^, insulin-like growth factor 1 receptor^20^, tyrosine-protein phosphatase non-receptor type 11^15^, and RAS p21 protein activator 1^21^. SOCS3 also causes anti-inflammatory effects by inhibiting JAK and signal transducer and activator of transcription (STAT) signal transduction. SOCS3 can affect a broad range of cytokines, especially when cytokines utilize JAK1, JAK2, or tyrosine kinase 2, or as long as SOCS3 binds to cytokine receptors^22^,^23^,^24^.

The levels of the proinflammatory cytokines IL-6 and tumor necrosis factor (TNF)-α are usually elevated during pathogen infections, and they induce pathological inflammation^25^,^26^. SOCS3 plays a role in regulating proinflammatory TNF-α signal transduction, leading to the silent progression of acute hepatitis C virus infection to chronicity^27^. TNF-α and IL-6 induce SOCS3 expression, and SOCS3 plays an important role in the negative feedback of some proinflammatory signal transducers such as TNF-α and IL-6^28^. TNF-α and IL-6 induce nuclear factor-kappa-B (NF-κB) inhibitor alpha (IκB-α) degradation and the translocation of NF-κB into the nucleus^29^,^30^,^31^. SOCS3, IL-6, and IL-8 expression is induced by TNF-α. TNF-α activates NF-κB signaling; thus, after translocating to the nucleus, NF-κB binds to the promoters of these genes and initiates their transcription^30^. In the present study, we found that the expression levels of SOCS3, IL-6, and TNF-α in PBMCs differed between piglets with PMWS and subclinical PCV2-infected piglets, and that SOCS3 plays an important role in regulating proinflammatory cytokines in subclinical PCV2 infections.

## Results

### The level of SOCS3, but not those of proinflammatory cytokines, is elevated in PBMCs from piglets with subclinical PCV2 infections, compared with PMWS piglets

**Figure 1:**
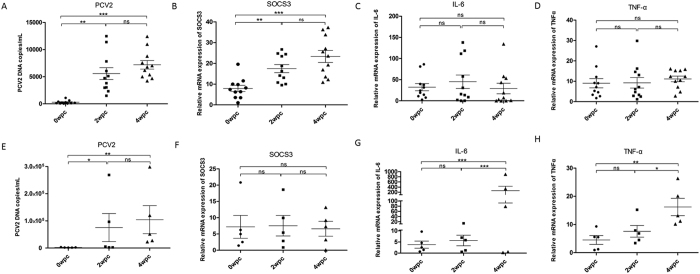
SOCS3 levels, but not proinflammatory cytokine levels, are elevated in PBMCs in subclinical PCV2-infected piglets, compared with PMWS piglets. (**A**) Viral loads of PBMCs from subclinical PCV2-infected piglets at 0–4 weeks post-challenge were measured by real-time RT-PCR. Endogenous SOCS3 (**B**), IL-6 (**C**), and TNF-α (**D**) mRNA levels were measured in PBMCs from subclinical PCV2-infected piglets at 0, 2, and 4 weeks post-challenge by real-time RT-PCR (n = 11). (**E**) Viral loads of PBMCs separated from PMWS piglets at 0–4 weeks. Endogenous SOCS3 (**F**), IL-6 (**G**), and TNF-α (**H**) mRNA levels were measured in PBMCs from PMWS piglets (n = 5). Statistical data were analyzed by paired *t*-tests and one-way analysis of variance (*P < 0.05; **P < 0.01; and ***P < 0.001; ns, not significant (*P* > 0.05)). All data are expressed as the mean ± SD.

Four weeks after challenge with PCV2, five piglets displayed obvious clinical symptoms, such as weight loss, mild pyrexia, rough hair coat, lethargy, and PCV2 antigen in their lymph nodes. The piglets were diagnosed with PMWS. Eleven piglets without significant clinical symptoms, but with PCV2 in their serum or lymph nodes, at 4 weeks post-challenge were diagnosed with PCV2 subclinical infections (data not shown). All the piglets had a significantly increased PCV2 load in their PBMCs, as determined by a quantitative polymerase chain reaction (qPCR) analysis (*P* < 0.05) (Fig. 1A,E).

Endogenous SOCS3 mRNA levels significantly increased in the PBMCs at 2 and 4 weeks post-challenge in the piglets in the PCV2 subclinical infection (group A2) (*P* < 0.01) (Fig. 1B), which differed significantly from those in the PMWS group A1 (Fig. 1F). The levels of IL-6 and TNF-α mRNAs in the PCV2 subclinical infection group did not change significantly compared to those at 0 weeks post-challenge (Fig. 1C,D). In the PMWS group, the IL-6 and TNF-α mRNA levels in PBMCs at 4 weeks post-challenge were significantly higher than those at 0 and 2 weeks (*P* < 0.05) (Fig. 1G,H).

### Proinflammatory signaling is suppressed in PBMCs from subclinical PCV2-infected piglets

**Figure 2:**
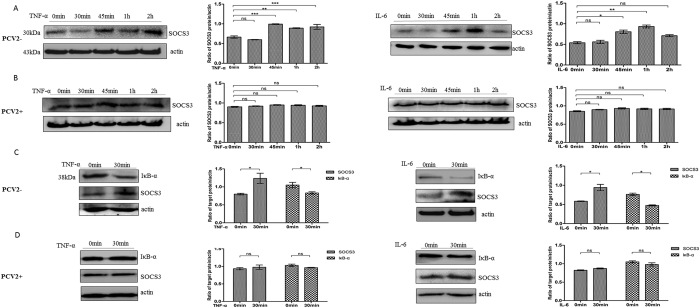
PCV2 inhibits SOCS3 expression and NF-κB signaling induced by TNF-α and IL-6 in PBMCs from PCV2-infected piglets. PBMCs separated from (**A**) healthy (n = 5) and (**B**) PCV2-infected piglets (n = 5) were treated with TNF-α (50 ng/ml) or IL-6 (100 ng/ml) for 0, 30, 45, 60, and 120 min. At each time point, PBMCs were lysed, and SOCS3 expression was analyzed by immunoblotting. PBMCs from (**C**) healthy (n = 5) and (**D**) PCV2-infected piglets (n = 5 of 11) were treated with TNF-α or IL-6 for 0 and 30 min. IκB-α protein levels were measured by immunoblotting. The intensities of the bands in the protein lysates were normalized to that of β-actin. Statistical data were analyzed by one-way analysis of variance (*P < 0.05; **P < 0.01; and ***P < 0.001; ns, not significant (*P* > 0.05)). All data are expressed as the mean ± SD. The samples were derived from the same experiment, and the blots of actin and IκB-α were processed in parallel. The blots of SOCS3 were performed in another gel using the same concentrations of the same samples. The two gels were run under the same conditions.

PBMCs were collected from the PCV2 subclinical infection group and healthy piglets (free of PCV2), then SOCS3 protein levels were detected following TNF-α (50 ng/ml) and IL-6 (200 ng/ml) stimulation. The SOCS3 protein was highly induced by 45 min of TNF-α or IL-6 treatment in PBMCs from healthy piglets (Fig. 2A) (P < 0.05), but not in PBMCs from subclinically infected piglets (Fig. 2B). This indicates that PCV2 may inhibit IL-6 and TNF-α signal transduction in PBMCs in subclinical infections.

PCV2 significantly inhibited the induction of NF-κB signaling by TNF-α and IL-6 (Fig. 2C,D). TNF-α and IL-6 induced IκB-α degradation in PBMCs from healthy piglets (*P* < 0.05). In contrast, IκB-α degradation was inhibited in PBMCs from subclinical PCV2-infected piglets following stimulation with TNF-α and IL-6, indicating that PCV2 blocked these two signaling pathways. These results suggest that PCV2 could inhibit signal transduction by proinflammatory cytokines and their induction of NF-κB signaling in PBMCs during subclinical infections.

### SOCS3 expression is elevated in PCV2-infected PK-15 porcine kidney cells

**Figure 3:**
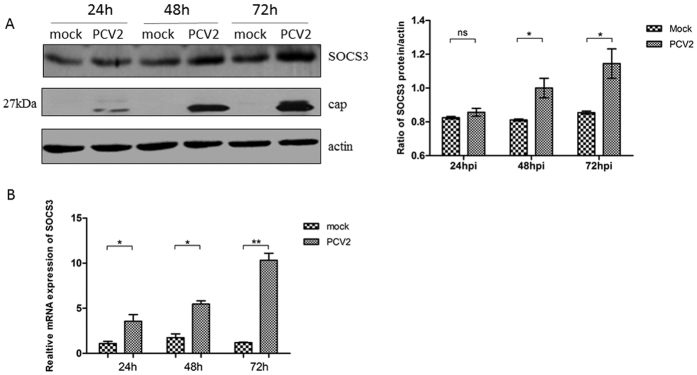
SOCS3 expression is elevated in PCV2-infected PK-15 cells. PK-15 cells were infected with the PCV2-SH strain at a MOI of 0.1 for 24, 48, and 72 h. At each time point, PK-15 cells were lysed and RNA was extracted. (**A**) Protein expression and (**B**) mRNA levels of endogenous SOCS3 were analyzed. The mRNA and protein expression levels of PCV2-infected cells are presented relative to the mRNA and protein expression levels of uninfected cells. The band intensity was normalized to that of β-actin. Each experiment was performed three times, and the results are representative of one independent experiment. Statistical data were analyzed by one-way analysis of variance (*P < 0.05; **P < 0.01; and ***P < 0.001; ns, not significant (*P* > 0.05)). All data are expressed as the mean ± SD. The samples were derived from the same experiment, and the blots of actin and SOCS3 were processed in parallel in the same gel. The blots of the PCV2 capsid protein were performed in another gel because the bands corresponding to SOCS3 and the capsid protein were too close to differentiate. The samples for blotting were prepared at the same concentrations, and the two gels were run under the same conditions.

PK-15 cells were infected with the PCV2-SH strain at a multiplicity of infection (MOI) of 0.1 for 24, 48, and 72 h. Endogenous SOCS3 mRNA and protein expression levels were detected in PCV2-infected PK-15 cells at 24, 48, and 72 h post-infection and compared with those in uninfected cells. SOCS3 mRNA and protein expression levels were significantly increased in PCV2-infected cells at 72 h post-infection (*P* < 0.05), indicating that SOCS3 expression was elevated by PCV2 infection in PK-15 cells (Fig. 3A,B).

### Overexpressed SOCS3 transiently supports PCV2 replication in PK-15 cells

**Figure 4:**
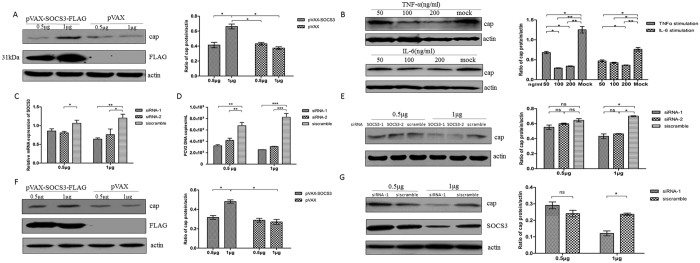
Overexpressed SOCS3 transiently supports PCV2 replication. (**A**) PK-15 cells were transfected with pVAX-SOCS3-FLAG at 0.5 and 1 μg, with pVAX used as a control. At 24 h post-transfection, treated cells were infected by PCV2 at 0.05 MOI and incubated for 36 h. Cells were lysed for Western blotting analysis of SOCS3 and PCV2 capsid protein. PCV2 capsid protein expression in infected cells is presented relative to that in mock-infected cells. (**B**) PK-15 cells were grown in 24-well plates and reached 90% confluence, infected by PCV2 at 0.1 MOI, and incubated for 24 h. Uninfected cells acted as mock-infected cells. At 24 h post-infection, recombinant TNF-α or IL-6 (50, 100 and 200 μg/ml) were added to PCV2-infected PK-15 cells for a further 12 h cultivation. Cells were lysed to analyse PCV2 capsid protein expression stimulated with TNF-α or IL-6. PK-15 cells were transfected with siRNASOCS3-1, siRNASOCS3-2, and siRNA scramble control at 0.5 or 1 μg (n = 4), and incubated for 48 h. Cells were infected by 0.05 MOI PCV2. At 36 h post-infection, cells were harvested for (**C**) mRNA expression analysis of RNA interference efficacy of SOCS3. (**D**) Culture supernatant was harvested and PCV2 DNA were detected by real-time PCR in the same sample. (**E**) Cell lysates were taken for analysis of PCV2 capsid protein expression. (**F**) 1 μg PCV2 infectious circular genome was co-transfected with pVAX-SOCS3-FLAG or pVAX (0.5 and 1 μg) into PK-15 cells. (G) 1 μg PCV2 infectious circular genome was co-transfected with siRNA-1 or siRNA scramble (0.5 and 1 μg) into PK-15 cells. After incubated for 60 h, the cells were lysed for Western blotting analysis of SOCS3 and capsid protein. All protein lysate band intensities were normalized to that of β-actin. Statistical data were analysed by one-way analysis of variance (*P < 0.05, **P < 0.01, and ***P < 0.001, ns represents non-significant *P* > 0.05). All data are expressed as the mean ± SD. The blots of actin and cap were processed in parallel in the same gel. The blots detecting SOCS3 were processed in another gel of the same samples prepared at the same concentrations.

To establish the relationship between SOCS3 and PCV2, PK-15 cells were transfected with 0.5 and 1 μg of pVAX-SOCS3-FLAG; pVAX was used as a control. At 24 h post-transfection, the cells were infected with the PCV2-SH strain at a MOI of 0.05, and at 36 h post-infection, the cells were collected for a Western blotting analysis of SOCS3 and the PCV2 capsid protein. Overexpression of SOCS3 (transfection with 1 μg of plasmid DNA) significantly increased PCV2 replication (*P* < 0.05) (Fig. 4A). Meanwhile, the same result was obtained when 1 μg of an infectious PCV2 circular genome was co-transfected with pVAX-SOCS3-FLAG, as shown in Fig. 4F.

To establish the relationship between proinflammatory cytokines and PCV2, PK-15 cells were infected by the PCV2-SH strain at a MOI of 0.1. After infection for 24 h, different concentrations of recombinant TNF-α or IL-6 were added to the PCV2-infected PK-15 cells, which were further cultivated for 12 h. Then, the cells were lysed and analyzed by Western blotting to detect the PCV2 capsid protein. The addition of 100 ng/ml TNF-α or IL-6 both significantly suppressed PCV2 replication in PK-15 cells (*P* < 0.05) (Fig. 4B).

To verify the above results, two small interfering RNA (siRNA) fragments (siRNA-1 and siRNA-2) of SOCS3 were transfected into PK-15 cells at 0.5 and 1 μg. A scrambled siRNA fragment was used as a control in a mock transfection. After the transfection with the SOCS3 siRNAs, the cells were infected with the PCV2-SH strain at a MOI of 0.05. At 36 h post-infection, the cells were collected to analyze the efficacies of the SOCS3 siRNAs (Fig. 4C). The culture supernatant was collected to determine the viral load (Fig. 4D), and the cells were lysed to analyze PCV2 capsid protein expression (Fig. 4E). Our results showed that PCV2 capsid expression and the viral load were obviously decreased when SOCS3 mRNA expression was knocked down following transfection with 1 μg of the siRNAs. Meanwhile, the same experiment was performed using 1 μg of an infectious PCV2 circular genome, which was co-transfected into the cells with siRNA-1. The results showed that the capsid expression level in the siRNA-1-treated cells (1 μg) was significantly lower than that in the scrambled siRNA control group (*P* < 0.05) (Fig. 4G).

### IL-6 and TNF-α expression is highly induced by PCV2 when SOCS3 is silenced *in vitro*

**Figure 5:**
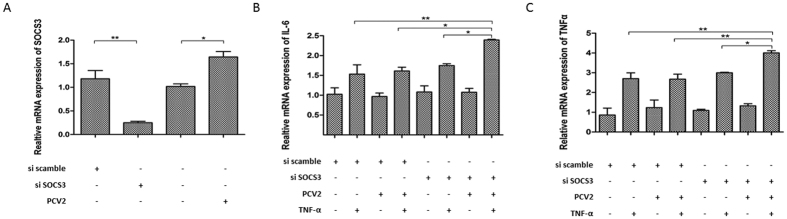
IL-6 and TNF-α expression is highly induced by PCV2 when SOCS3 is silenced *in vitro.* (**A**) PK-15 cells were grown in 24-well plates until they reached 90% confluence. Cells were transfected with SOCS3 siRNAs or the scrambled siRNA control for 24 h. At 24 h post-transfection, cells were infected with the PCV2-SH strain, or not, at a MOI of 0.1 for 24 h before stimulation with TNF-α for a further 12 h. Mock-infected cells served as a control. The SOCS3 mRNA level in PK-15 cells was measured by qPCR (n = 3). At 24 h post-infection, TNF-α at 100 ng/ml was added to the culture supernatant for a further 12 h. IL-6 (**B**) and TNF-α (**C**) mRNA levels in PK-15 cells were measured by qPCR (n = 3). Relative SOCS3, IL-6, and TNF-α gene expression was compared with that of the scrambled siRNA control. Statistical data were analyzed by one-way analysis of variance (*P < 0.05; **P < 0.01; and ***P < 0.001; ns, not significant (*P* > 0.05)). All data are expressed as the mean ± SD.

To discover the roles of PCV2 and SOCS3 in regulating cytokines *in vitro*, PK-15 cells were transfected with the SOCS3 siRNAs or a scrambled siRNA control, and then SOCS3 mRNA was analyzed by qPCR at 24 h post-transfection. The relative expression of silenced SOCS3 was reduced to one-third of that of the scrambled siRNA control (Fig. 5A). Then, the cells were infected with the PCV2-SH strain, or not, at a MOI of 0.1 for 24 h before stimulation with TNF-α for a further 12 h. At 24 h post-infection, PCV2 infection induced SOCS3 mRNA expression (Fig. 5A). The IL-6 level is known to be upregulated by TNF-α; thus, TNF-α was added to the culture supernatant at 100 ng/ml for a further 12 h, and RNA was extracted from cells to detect the IL-6 and TNF-α levels by qPCR. At 36 h post-infection, the qPCRs showed that TNF-α induced IL-6 expression, and higher IL-6 and TNF-α levels were induced by PCV2 when SOCS3 was silenced, compared with control cells (Fig. 5B,C). This demonstrates that SOCS3 plays an important role in inhibiting IL-6 and TNF-α levels during PCV2 infection *in vitro*.

### SOCS3 reduces the induction of NF-κB signaling by TNF-α and IL-6 *in vitro*

**Figure 6:**
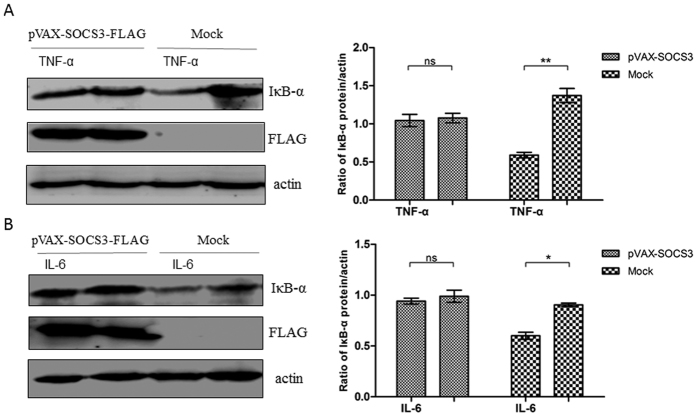
SOCS3 inhibits the induction of NF-κB signaling by TNF-α and IL-6 *in vitro.* PK-15 cells were transfected with pVAX-SOCS3 (1 μg) or pVAX (1 μg). At 24 h post-transfection, the cells were treated with TNF-α (50 ng/ml) or IL-6 (200 ng/ml) for 20 min; non-stimulated cells served as a control. Then, the cells were lysed and subjected to western blotting to analyze IκB-α degradation following stimulation with (**A**) TNF-α or (**B**) IL-6. IκB-α protein expression is presented relative to that of β-actin. Statistical data were analyzed by one-way analysis of variance (*P < 0.05; **P < 0.01; and ***P < 0.001; ns, not significant (*P* > 0.05)). All data are expressed as the mean ± SD. The samples were derived from the same experiment, and the blots of IκB-α and actin were processed in parallel in the same gel. The blots of SOCS3-FLAG were performed in another gel using the same samples because the bands of SOCS3-FLAG and IκB-α were too close to differentiate. The samples for blotting were prepared at the same concentrations, and the two gels were run under the same conditions.

TNF-α and IL-6 induced IκB-α degradation in PBMCs from healthy piglets, whereas IκB-α degradation was inhibited in PBMCs from PCV2-infected piglets. To discover whether this also occurred in PK-15 cells, PK-15 cells were transfected with pVAX-SOCS3 or pVAX. IκB-α degradation was detected following TNF-α (50 ng/ml) or IL-6 (200 ng/ml) stimulation. IκB-α degradation was highly induced by TNF-α or IL-6 in mock-transfected cells, but it was inhibited in PK-15 cells that overexpressed SOCS3 (Fig. 6A,B), indicating that SOCS3 could block NF-κB signaling pathways. These results suggest that SOCS3 expression inhibits the induction of NF-κB signaling by proinflammatory cytokines in PK-15 cells.

### SOCS3 interacts with STAT3 and TNF-receptor-associated factor (TRAF) 2

**Figure 7:**
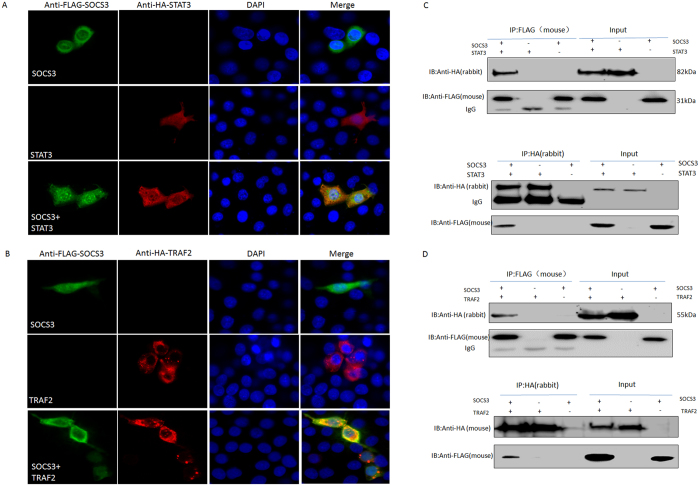
SOCS3 interacts with STAT3 and TRAF2. pVAX-SOCS3-FLAG and pVAX-STAT3-HA (**A**) or pVAX-TRAF2-HA (**B**) were transfected or co-transfected into PK-15 cells. At 24 h post-transfection, the cells were fixed in 4% paraformaldehyde for 30 min. After blocking with 1% bovine serum albumin, the cells were incubated with anti-HA and anti-FLAG antibodies at a 1:200 dilution. Alexa Fluor 594-conjugated goat anti-rabbit IgG and Alexa Fluor 488-conjugated goat anti-mouse IgG were mixed at a 1:400 dilution. The cells were examined by laser-scanning confocal microscopy. PK-15 cells were co-stained with a FLAG-specific mouse monoclonal antibody (green) and an HA-specific rabbit polyclonal antibody (red). Nuclei were stained using DAPI (blue). After co-transfection with these plasmids and incubation for 24 h, the cells were collected and lysed, and SOCS3 was immunoprecipitated using the anti-FLAG (mouse) antibody. Immunoblotting was performed using the anti-HA (rabbit) and anti-FLAG (mouse) antibodies. Whole-cell lysates were also analyzed for SOCS3 and STAT3 (**C**) or TRAF2 (**D**) expression. Bands corresponding to SOCS3, STAT3, TRAF2, and IgG are indicated. A reverse immunoprecipitation experiment was performed using the anti-HA (rabbit) antibody to bind STAT3-HA and TRAF2-HA. Immunocomplexes were detected by immunoblotting using antibodies against the HA (mouse or rabbit) and FLAG (mouse) epitopes.

STAT3 and TRAF2 have important roles in IL-6 and TNF-α signaling, respectively. To understand the relationship between SOCS3 and STAT3 or TRAF2 in PK-15 cells, their expression was observed by confocal microscopy. Hemagglutinin-tagged STAT3 (STAT3-HA) (red) and FLAG-tagged SOCS3 (SOCS3-FLAG) (green) were mainly distributed in the cytoplasm of PK-15 cells. When PK-15 cells were co-transfected with plasmids expressing SOCS3 and STAT3, co-localization of SOCS3 and STAT3 was observed in PK-15 cells (Fig. 7A). TRAF2-HA (red) also co-localized with SOCS3- FLAG (green) (Fig. 7B).

To further investigate the interaction of SOCS3 with STAT3 or TRAF2, pVAX-STAT3-HA and pVAX-SOCS3-FLAG or pVAX-TRAF2-HA and pVAX-SOCS3-FLAG were co-transfected into PK-15 cells. Cell lysates were immunoprecipitated with mouse monoclonal antibodies against the FLAG epitope or with rabbit polyclonal antibodies against the HA epitope. Proteins in the immune complexes were analyzed by western blotting with anti-HA and anti-FLAG antibodies. STAT3-HA and TRAF2-HA were detected in immunoprecipitates obtained with the anti-FLAG antibody (Fig. 7C,D), and SOCS3-FLAG was immunoprecipitated with the anti-HA antibody, indicating the specificity of the interaction. These data indicate that SOCS3-FLAG interacted with STAT3-HA and TRAF2-HA in PK-15 cells, supporting the hypothesis that this interaction resulted in the co-localization of SOCS3 with STAT3 and TRAF2, and indicating that SOCS3 plays a role in inhibiting IL-6 and TNF-α signaling by anchoring STAT3 and TRAF2.

## Discussion

PCV2 infections are widespread in the swine industry^32^,^33^. Among PCV2-endemic herds, only 4–20% of pigs exhibit clinical signs of PMWS^34^. It is suggested that PCV2 evokes latent subclinical symptoms without any obvious signs of infection^35^. The mechanism that triggers subclinical symptoms is not fully understood. The putative pathogenic mechanism may be associated with inflammatory reactions, and it is necessary to search for the host factors that are involved in latent (subclinical) PCV2 infections. In this study, a challenge experiment showed that SOCS3 expression levels were significantly increased in PBMCs from subclinical piglets, but not in those from PMWS piglets. Meanwhile, the expression of the proinflammatory cytokines IL-6 and TNF-α increased in PBMCs from piglets with PMWS, as described previously^10^,^11^, but not in those from subclinical PCV2-infected piglets. Additionally, the proinflammatory signaling and its induced NF-κB signaling were suppressed in PBMCs from subclinical PCV2-infected piglets, although further studies will be needed to determine whether this occurs over longer time periods, e.g., 3 or 6 months post-infection. Furthermore, *in vitro* results showed that SOCS3 bound to STAT3 and TRAF2 in PK-15 cells, which offers a possible mechanism by which SOCS3 inhibits proinflammatory cytokine signaling. The results suggest that SOCS3 plays an important role in regulating proinflammatory cytokines in subclinical PCV2 infections. However, the relationship between SOCS3 and PCV2 that was observed in the *in vitro* infections may not mirror that in subclinical infections *in vivo*. Further studies should be focused on this point.

SOCS3 is associated with inflammatory reactions, and it acts as a negative regulator of Th17-cell differentiation, which is involved in inflammatory diseases^36^. SOCS3 is the key regulator of proinflammatory cytokines, such as IL-6 and TNF-α, and anti-inflammatory cytokines such as IL-10. Selective binding to its receptor, GP130, determines whether the regulatory function of SOCS3 has proinflammatory or anti-inflammatory effects^37^. SOCS3 directly or indirectly interacts with microbial pathogens through a variety of signaling pathways, including JAK–STAT and NF-κB, as a consequence of the host immune response. For example, hepatitis B virus-induced SOCS1 and SOCS3 expression explains why patients with high viral loads do not respond well to IFN treatment^38^. Influenza-induced SOCS1 may exacerbate lung injury^39^. PBMCs from PCV2-infected piglets have an increased IL-6 level. PMWS is also associated with increases in IL-6, IFN-γ, and TNF-α mRNA levels in white blood cells^10^,^11^. Borghetti *et al*.^40^ concluded that the low levels of proinflammatory cytokines, such as IL-8 and TNF-α, in PMWS-infected animals were responsible for the low innate proinflammatory response to PCV2 infection. These low innate proinflammatory responses failed to cope with PCV2 infection, while high levels of proinflammatory cytokines can aggravate PCV2 phlegmonosis in piglets with PMWS^10^,^11^. Thus, we presumed that PCV2 infection may manipulate SOCS3 to escape the host immune response, thereby leading to a subclinical infection. Our *ex vivo* results showed that piglets with subclinical PCV2 infections expressed proinflammatory cytokines at basal levels, which differed from those in the PBMCs from pigs with PMWS. The induction of SOCS3 expression by PCV2 suppresses the inflammatory response to TNF-α and IL-6 in PBMCs from PCV2-infected piglets. This suggests that suppression of the inflammatory reaction might explain the progression of PCV2 latency, as well as the relatively mild and slow progress of PCV2 pathogenesis in piglets.

PBMCs from PCV2-infected piglets with PMWS show increased IL-2, IL-4, IL-6, and IL-10 levels^10^,^11^, and PK-15 cells infected with PCV2 produce IL-6, according to Choi *et al*.^41^. This indicates that the upregulation of proinflammatory cytokines in infected pigs may contribute to PCV2 pathogenesis. In the present study, the *in vitro* results showed that PCV2 induced SOCS3 expression in PK-15 cells. Additionally, PCV2 infection of PK-15 cells that were treated with SOCS3 siRNAs exhibited elevated levels of IL-6 and TNF-α, compared with control cells, thereby demonstrating that PCV2 activated SOCS3 to minimize these proinflammatory responses to promote a subclinical infection. Meanwhile, PCV2 increased SOCS3 expression *in vitro*, which also inhibited the IL-6- or TNF-α-mediated activation of the NF-κB signaling pathway, leading to decreased synthesis or activation of cytokine components. In addition, overexpression of SOCS3 inhibited IL-6 and TNF-α expression, and recombinant IL-6 and TNF-α decreased PCV2 replication in PK-15 cells. These results suggest that PCV2 could survive and avoid inducing strong inflammatory reactions by enhancing SOCS3 expression. Of course, we note that SOCS3 expression enhances a productive PCV2 infection *in vitro*. However, this does not explain acute infections in animals, in which more PCV2 DNA copies were detected in PBMCs from PMWS animals, while there was no change in SOCS3 expression following virus challenge. The difference in SOCS3 expression and proinflammatory cytokine production in PBMCs needs to be investigated further in normal control piglets and sick animals with or without symptoms.

The TRAF protein family is associated with the TNF receptor superfamily, and it plays an important role in signal transduction. TRAF directly interacts with TNF receptors, and it is required for TNF-α-mediated activation of mitogen-activated protein kinase 8/C-Jun N-terminal kinase and NF-κB^16^,^17^,^18^. TNF-α signals are closely connected to TRAF and a TNF-receptor-associated death domain protein^42^,^43^,^44^. GP130 is the receptor of IL-6, and JAK and GP130 interact with SOCS3; the latter interaction inhibits IL-6 signal transduction. STAT3 plays an important role in mediating IL-6 signaling^45^. SOCS3 inhibits STAT3 phosphorylation by binding to the IL-6 family of cytokines^46^, and then it reduces STAT activity and exerts an anti-inflammatory effect. TRAF2 is also a key component in TNF-α signaling that interacted with SOCS3 in our study, in accordance with a study by Collins *et al*.^27^. Our results confirmed the co-localization of SOCS3 with STAT3 and TRAF2 in PK-15 cells, indicating a possible role of SOCS3 in inhibiting IL-6 and TNF-α signaling by anchoring STAT3 and TRAF2. However, the same study should be performed on PBMCs from subclinical PCV2-infected pigs in the future.

In conclusion, our findings reveal a novel mechanism by which PCV2 induces SOCS3 expression by interacting with STAT3 and TRAF2 in PBMCs and PK-15 cells to regulate proinflammatory signaling. These results will aid our understanding of why subclinical PCV2 infections result in mild pathogenesis in susceptible organs, although the mechanism by which PCV2 enhances the expression of SOCS3 is still unknown and requires further study.

## Materials and Methods

### Materials

The PCV2-SH strain, which was isolated by our laboratory, belongs to PCV type 2b (GenBank accession no. AY686763). Plasmid pEasy-PCV2b containing the complete genome of PCV2-SH was prepared by our laboratory. PK-15 cells (American Type Culture Collection, Manassas, VA, USA) were grown in Dulbecco’s modified Eagle’s medium supplemented with 10% fetal calf serum, 250 U/ml penicillin, and 250 μg/ml streptomycin. PBMCs were grown in Roswell Park Memorial Institute 1640 medium supplemented with 10% fetal calf serum, 1% non-essential amino acids, 250 U/ml penicillin, and 250 μg/ml streptomycin, and they were cultured at 37 °C in 5% CO_2_. SOCS3 was amplified from PK-15 cells and inserted into plasmid pVAX such that it was fused to a FLAG epitope tag. TRAF2 and STAT3 were amplified from PK-15 cells and inserted into plasmid pVAX such that they were fused to an HA epitope tag. The primer sequences are as follows: SOCS3-F, 5′–GCGGATCCATGGTCACCCACAGCAAGT–3′; SOCS3-R-FLAG, 5′–CGGAATTCTTACTTATCGTCGTCATCCTTGTAATCAA GTGGGGCATCGTA–3′; TRAF2-F, 5′–CGGGATCCATGGCTGCAGCCAGCGTGA–3′; TRAF2-R-HA, 5′–CCGAATTCCTAAGCGTAGTCTGGGACGTCGTATGGGTA GAGCCCCGTCAGGTCCACG–3′; STAT3-F, 5′–GCAAGCTTATGGCCCAATGGA ATCAG–3′; STAT3-R-HA, 5′–GCGAATTCTCAAGCGTAGTCTGGGACGTCGTAT GGGTACATGGGGGAGGTAGCG–3′.

TNF-α and IL-6 were used at 50 and 200 ng/ml, respectively (Sino Technology, USA). SOCS3 and scrambled control siRNA fragments were transfected into PK-15 cells using Lipofectamine 3000^TM^ (Invitrogen, Carlsbad, CA, USA) according to the manufacturer’s instructions. IL-6 and TNF-α mRNAs in the cells were quantified by qPCR. Protein A+G for the immunoprecipitation assay was purchased from Beyotime (Beyotime, Shanghai, China). Anti-β-actin, anti-SOCS3, and anti-HA antibodies were purchased from Santa Cruz Biotechnology (Dallas, TX, USA). The anti-FLAG antibody was purchased from Abmart (Shanghai, China), and the anti-PCV2 capsid antibody was prepared by our laboratory. The molecular weights of β-actin, SOCS3, TRAF2, STAT3, the PCV2 capsid protein, and IκB-α protein were approximately 43, 30, 55, 82, 27, and 38 kDa, respectively. 4′,6-diamidino-2-phenylindole (DAPI), Alexa Fluor 488-conjugated goat anti-mouse IgG, and Alexa Fluor 594-conjugated goat anti-rabbit IgG were purchased from Invitrogen. DNA and RNA were extracted from cells using appropriate kits (Omega, Norcross, GA, USA).

### Animal experiment

Twenty-one 3-week-old piglets free of PCV2 and porcine respiratory and reproductive syndrome virus were randomly divided into two groups and housed separately. The first group A (n = 16) were challenged as described previously^47^,^48^. Briefly, the pigs were inoculated intranasally with 5 × 10^5^ median tissue culture infective doses of the PCV2 SH strain per pig. At 4 and 7 d post-challenge, the pigs were injected at four sites (in each axilla and each hip) with 2 ml of a solution containing 2 mg of keyhole limpet hemocyanin (Sigma-Aldrich, St. Louis, MO, USA) emulsified in incomplete Freund’s adjuvant (Sigma-Aldrich) (0.5 ml per site), and they were also given an intraperitoneal injection of 10 ml of thioglycollate broth (glycan, Sigma-Aldrich). Then, the piglets received additional intraperitoneal injections of glycan at 11 and 19 d post-challenge. During the 4 weeks of observation, the piglets with or without PMWS -related clinical symptoms were divided into two separate groups (A1 and A2). Five other piglets were used as a healthy control group. PBMCs were separated from all of the piglets, and DNA and RNA were extracted to examine TNF-α, IL-6, SOCS3, and PCV2. At 4 weeks post-challenge, the piglets were sacrificed, diagnosed for PMWS by a pathological examination, and immunohistochemistry was used to detect the PCV2 antigen in lymph node samples. The PCV2 loads in PBMCs were detected by qPCR as described previously^7^,^49^.

All experimental protocols were approved by the Institutional Animal Care and Ethics Committee of Nanjing Agricultural University (Nanjing, Jiangsu, China) and met the standards of the International Guiding Principles for Biomedical Research Involving Animals.

### *Ex vivo* experiment

Blood (10 ml) was collected from each piglet every 2 weeks, and PBMCs were isolated by density gradient centrifugation using a lymphocyte isolation liquid as described previously^50^. Then, the cells were washed twice with Roswell Park Memorial Institute 1640 medium, diluted, and cultured in a 24-well plate at a concentration of 1 × 10^6^ cells per well. After culturing for 12 h, all of the cells were collected and total RNA was extracted. Then, 500 ng of RNA per sample was used to synthesize cDNA to detect proinflammatory cytokine or SOCS3 mRNAs in the PBMCs from each piglet. In addition, for those cells that were stimulated by cytokines, TNF-α (50 ng/ml) and IL-6 (200 ng/ml) were added to the supernatant, and the cells were cultured for 0, 30, 45, 60 and 120 min. Then, the cells were lysed by lysis buffer prior to western blotting.

### *In vitro* experiment

To determine the relationship between SOCS3 and PCV2 *in vitro*, PK-15 cells were counted, diluted to 1 × 10^6^ per well, and grown in a 24-well plate for 12 h. PK-15 cells were transfected with plasmid pVAX-SOCS3-FLAG at 0.5 and 1 μg; pVAX plasmid was used as a control. At 24 h post-transfection, the cells were infected with the PCV2 SH strain at a MOI of 0.05. After incubation for a further 36 h, the cells were lysed for a western blotting analysis of SOCS3 and the PCV2 capsid protein. Meanwhile, 1 μg of an infectious PCV2 circular genome was prepared according to Hua *et al*.^51^, and it was co-transfected into PK-15 cells with pVAX-SOCS3-FLAG or pVAX at doses of 0.5 and 1 μg. After incubation for 60 h, the cells were lysed for a western blotting analysis of SOCS3 and the PCV2 capsid protein.

To analyze the efficacies of siRNAs targeting to SOCS3, PK-15 cells were transfected with 0.5 or 1 μg of two siRNAs (siRNA-1 and siRNA-2) or a scrambled siRNA control. After incubation for 48 h, the cells were infected with the PCV2 SH strain at a MOI of 0.05. At 36 h post-infection, the cells were lysed for qPCR and western blot analyses. Meanwhile, 1 μg of an infectious PCV2 circular genome was co-transfected with siRNA-1 or the scrambled siRNA (at doses of 0.5 and 1 μg) into PK-15 cells. After incubation for 60 h, the cells were lysed for a western blotting analysis of SOCS3 and the PCV2 capsid protein.

To determine the relationship between cytokines and PCV2 *in vitro*, PK-15 cells were grown in 24-well plates until they reached 90% confluence. Then, they were infected by the PCV2-SH strain at a MOI of 0.1 and incubated for 24 h. Mock-infected cells served as a control. At 24 h post-infection, recombinant TNF-α or IL-6 (50, 100 and 200 μg/ml) was added to the PCV2-infected PK-15 cells, followed by a further 12 h cultivation, and the cells were lysed to analyse PCV2 capsid protein expression.

For NF-κB signaling detection *in vitro*, PK-15 cells were transfected with 1 μg of pVAX-SOCS3 or pVAX. At 24 h post-transfection, the cells were treated with TNF-α (50 ng/ml) or IL-6 (200 ng/ml) for 20 min, lysed, and IκB-α degradation was analyzed by western blotting.

### Quantitative reverse transcription-PCR (qRT-PCR)

Total DNA from PBMCs was isolated following the manufacturer’s instructions (Omega, Norcross, GA, USA), and assayed by real-time PCR. Total RNA was extracted from the cellular samples using an extraction kit. Reverse transcription was conducted using Moloney murine leukemia virus reverse transcriptase (Promega, Madison, WI, USA) according to the manufacturer’s instructions. Two microliters of the RT reaction mixture was subjected to qRT-PCR using specific primers for SOCS3 (F: 5′–GCCCCC CTAGAAGAGCCTATTA–3′ and R: 5′–TCCGACAGAGATGCTGGAG A–3′), IL-6 (F: 5′–ATGAGAAGTGTGAAAACAGCAAGG–3′ and R: 5′–CATTTGTGGTGGGGTTA GGG–3′), PCV2 (F: 5′–CCAGGAGGGCGTTCTGACT–3′, PCV2-RT-R: 5′–CGTTACC GCTGGAGAAGGAA–3′, PROBE: 5′–FAM-AATGGCATCTTCAACACCCGCCTC-TARAM–3′), β-actin (F: 5′–CTCTTCCAGCCCTCCTTCCT–3′ and R: 5′–ACGT CGCACTTCATGATCGA–3′). The SYBR Green Real-time PCR Mix (Vazyme, Nanjing, China) was used according to the manufacturer’s recommendations. The reaction procedure was as follows: 95 °C for 5 min, followed by 40 cycles at 95 °C for 10 s and 60 °C for 31 s. Data analysis was conducted using the 2^−ΔΔCt^ comparative threshold method, and gene expression was normalized to the level of β-actin mRNA. The qRT-PCR was performed on an ABI PRISM 7300 (Applied Biosystems, Foster City, CA, USA).

### Western blot analysis

PK-15 cells were lysed, and the protein concentration was determined with the Pierce BCA Protein Assay Kit (Thermo Fisher Scientific, Waltham, MA, USA). Equivalent amounts of cell lysate protein (30 μg) were subjected to 10% sodium dodecyl sulfate–polyacrylamide gel electrophoresis and transferred to 0.22-μm nitrocellulose membranes (Pall, Port Washington, NY, USA). Then, the membranes were incubated with mouse monoclonal or rabbit polyclonal antibodies at 37 °C for 1 h. After washing three times with phosphate-buffered saline (PBS) containing 0.05% Triton X-100, the membranes were incubated at 37 °C for 1 h with horseradish peroxidase-conjugated goat anti-mouse IgG or goat anti-rabbit IgG. Detection was performed using chemiluminescence luminal reagents (Thermo Fisher Scientific).

### Immunoprecipitation

PK-15 cells were transfected with 1 μg of pVAX or pVAX-SOCS3-FLAG and pVAX-TRAF2-HA, or pVAX-SOCS3-FLAG and pVAX-STAT3-HA, in 24-well cell culture plates (Thermo Fisher Scientific) for 24 h. At 24 h post transfection, the cells were lysed in 100 μl of lysis buffer (Beyotime). Cell lysates were centrifuged (5,000 × *g*, 5 min), and the supernatants were incubated overnight at 4 °C with 1 μg of anti-FLAG or anti-HA antibodies, and then pre-coupled to 40 μl of A/G Plus Agarose beads for 4 h at 4 °C. The immune complexes were precipitated, washed, and subjected to a western blotting analysis.

### Confocal microscopy

PK-15 cells were transfected with 1 μg of pVAX or pVAX-SOCS3-FLAG and pVAX-TRAF2-HA, or pVAX-SOCS3-FLAG and pVAX-STAT3-HA, in 24-well cell culture plates for 24 h. At 24 h post transfection, the cells were processed for indirect immunofluorescence by fixation in 4% paraformaldehyde for 30 min. Cell membranes were permeabilized by PBS containing 0.2% Triton X-100 for 5 min, and then blocked with 1% bovine serum albumin for 30 min. The cells were incubated with anti-HA and anti-FLAG antibodies, both at a 1:200 dilution, at 37 °C for 1 h. After washing three times with PBS, Alexa Fluor 594-conjugated goat anti-rabbit IgG and Alexa Fluor 488-conjugated goat anti-mouse IgG mixed (at a 1:400 dilution) were added to the target well and incubated for an additional 1 h. After washing three times with PBS, cell nuclei were stained by DAPI for 5 min before examining them by laser-scanning confocal microscopy.

### siRNAs

RNA interference was performed by transfecting siRNA fragments of SOCS3 and a scrambled siRNA control. Two pairs of siRNAs were as follows: CS3-1, 5′–GGU CACCCACAGCAAGUUUTT–3′ and 5′–AAACUUGCUGUGGGUGACCTT–3′; CS3-2, 5′–CCUGGACUCCUAUGAGAAATT–3′ and 5′–UUUCUCAUAGGAGUCCAGGTT– 3′. The siRNA fragments were transfected into PK-15 cells at 0.5 μg and 1 μg using Lipofectamine 3000 (Invitrogen) according to the manufacturer’s instructions. The efficacy of RNA interference was evaluated by a qPCR analysis.

### Statistical analysis

Statistical analysis was performed using GraphPad Prism version 5.0.2 (GraphPad Software, San Diego, CA, USA). Comparisons between groups were performed using paired *t*-tests and one-way analysis of variance. *P* < 0.05 represents a statistically significant difference. All data are expressed as the mean ± standard deviation (SD).

## Additional Information

**How to cite this article**: Zhu, X. *et al*. Suppressor of cytokine signaling 3 plays an important role in porcine circovirus type 2 subclinical infection by downregulating proinflammatory responses. *Sci. Rep.* **6**, 32538; doi: 10.1038/srep32538 (2016).
